# Verification of a Portable Motion Tracking System for Remote Management of Physical Rehabilitation of the Knee

**DOI:** 10.3390/s19051021

**Published:** 2019-02-28

**Authors:** Kevin M. Bell, Chukwudi Onyeukwu, Michael P. McClincy, Marcus Allen, Laura Bechard, Abhigyan Mukherjee, Robert A. Hartman, Clair Smith, Andrew D. Lynch, James J. Irrgang

**Affiliations:** 1 Department of Orthopaedic Surgery, School of Medicine, University of Pittsburgh, Pittsburgh, PA 15213, USA; Onyeukwu.Chukwudi@medstudent.pitt.edu (C.O.); mcclincymp@upmc.edu (M.P.M.); leb94@pitt.edu (L.B.); rob.a.hartman@gmail.com (R.A.H.); CNS45@pitt.edu (C.S.); jirrgang@pitt.edu (J.J.I.); 2 Department of Bioengineering, Swanson School of Engineering, University of Pittsburgh, Pittsburgh, PA 15213, USA; 3 Clinical and Translational Science Institute, University of Pittsburgh, Pittsburgh, PA 15213, USA; 4 Department of Mechanical Engineering and Materials Science, Swanson School of Engineering, University of Pittsburgh, Pittsburgh, PA 15213, USA; mca36@pitt.edu; 5 Department of Physical Therapy, School of Health and Rehabilitation Sciences, University of Pittsburgh, Pittsburgh, PA 15213, USA; adl45@pitt.edu; 6 School of Medicine, University of Pittsburgh, Pittsburgh, PA 15213, USA; mukherab@rutgers.edu

**Keywords:** knee, rehabilitation, physical therapy, mobile health, mHealth, inertial measurement units

## Abstract

Rehabilitation following knee injury or surgery is critical for recovery of function and independence. However, patient non-adherence remains a significant barrier to success. Remote rehabilitation using mobile health (mHealth) technologies have potential for improving adherence to and execution of home exercise. We developed a remote rehabilitation management system combining two wireless inertial measurement units (IMUs) with an interactive mobile application and a web-based clinician portal (interACTION). However, in order to translate interACTION into the clinical setting, it was first necessary to verify the efficacy of measuring knee motion during rehabilitation exercises for physical therapy and determine if visual feedback significantly improves the participant’s ability to perform the exercises correctly. Therefore, the aim of this study was to verify the accuracy of the IMU-based knee angle measurement system during three common physical therapy exercises, quantify the effect of visual feedback on exercise performance, and understand the qualitative experience of the user interface through survey data. A convenience sample of ten healthy control participants were recruited for an IRB-approved protocol. Using the interACTION application in a controlled laboratory environment, participants performed ten repetitions of three knee rehabilitation exercises: heel slides, short arc quadriceps contractions, and sit-to-stand. The heel slide exercise was completed without feedback from the mobile application, then all exercises were performed with visual feedback. Exercises were recorded simultaneously by the IMU motion tracking sensors and a video-based motion tracking system. Validation showed moderate to good agreement between the two systems for all exercises and accuracy was within three degrees. Based on custom usability survey results, interACTION was well received. Overall, this study demonstrated the potential of interACTION to measure range of motion during rehabilitation exercises for physical therapy and visual feedback significantly improved the participant’s ability to perform the exercises correctly.

## 1. Introduction

Rehabilitation following knee injury or surgery is critical for recovery of function and independence—not just to regain strength, flexibility, and joint range of motion (RoM), but also to decrease pain, stiffness, and swelling [1]. Successful post-operative rehabilitation can minimize complications such as wound infection, deep vein thrombosis, and pulmonary embolism [2]. However, patient non-adherence remains a significant barrier to successful rehabilitation. Physical therapy non-adherence rates can reach up to 76% [3,4]. Rehabilitation non-adherence contributes to significant healthcare burdens such as: personal health costs, unnecessary health provider visits, prescription drug use, increased risk of complications, and decreased quality of life [1].

Barriers to adherence are multifactorial and a result of inadequate education or poor communication, patients frequently underappreciate the importance of rehabilitation. This is problematic as patients are expected to perform exercises independently for the majority of their recovery period [5]. Patients from low-income households may face bigger barriers to rehabilitation due to limited access to rehabilitation facilities, inability to handle copays, and low health literacy [6,7,8]. Accessibility is also a struggle for patients with large family or work responsibilities, who live alone, and/or who experience disabling amounts of pain [9,10]. Finally, psychological barriers, such as poor self-efficacy, fear avoidance behaviors, and distress can also prevent adherence [5].

Remote rehabilitation utilizing mobile health (mHealth) technologies has emerged as a potential method for improving adherence and promoting effective execution of home exercise [11,12]. A promising complement to conventional in-person therapy, mHealth technologies enable remote delivery of therapy interventions, and can monitor performance and adherence in real-time when combined with biofeedback [13]. Using mHealth technologies, health care providers can reduce barriers to accessibility and communication while also managing objective records of adherence and performance. 

Hussain et al. presented a clinical trial protocol for a mobile application and web-based tool to support the delivery of total knee replacement care [14]. Their system utilizes a wrist-worn activity tracker to monitor overall activity, as opposed to the IMU-based system presented in this manuscript, which tracks knee joint motion specifically. There have also been a few studies that have evaluated the use of the Nintendo Wii^®^ Fit^®^ console as an adjunct to conventional physical therapy [15,16,17]. More directly related to the approach utilized in this manuscript, Piqueras et al. evaluated the effectiveness of a virtual telerehabilitation system in patients after total knee replacement [18]. They determined that a two-week interactive telerehabilitation program was at least as effective as conventional therapy. Their system utilized nine degree-of-freedom wireless kinematic sensors. Similarly, Correia et al. presented a home-based rehabilitation platform that included digital biofeedback [19]. They completed an eight-week feasibility study demonstrating that their digital rehabilitation solution achieved better outcomes than conventional in-person rehabilitation. However, Piqueras et al. and Correia et al. did not provide data about the reliability sensors and the effectiveness of the biofeedback. 

Building upon this work, we have developed a novel remote rehabilitation management system (interACTION) combining two affordable, portable wireless inertial measurement units (IMUs) with an interactive mobile application and a web-based clinician portal. Real-time visual feedback provided by the IMUs permits self-monitoring and adjustment during exercise for patients, while the clinician portal (not evaluated in the present study) enables the clinician to monitor adherence and recovery remotely. 

In order to translate interACTION into the clinical setting, it is first necessary to verify the efficacy of measuring knee RoM during rehabilitation exercises for physical therapy and to determine if visual feedback significantly improves the participant’s ability to perform the exercises correctly. Therefore, the first aim of this study was to verify the accuracy of the IMU-based knee angle measurement system by comparing motion tracking of the IMU-based system with a video-based motion tracking system during three common physical therapy exercises. The second aim of this study was to quantify the effect of visual feedback on exercise performance. Beyond assessing the efficacy of the technology, another important component to clinical translation is to determine if the intended users are willing and able to wear and use the technology required for the intervention. Therefore, the third aim of this study was to evaluate the wearability, usability, and overall look and feel of the technology using a custom survey that was administered at the conclusion of the testing session. Participants also completed a validated measure of user satisfaction for ease of use, amount of time needed, and clarity of instruction. 

## 2. Materials and Methods

### 2.1. IMU Motion Tracking Sensor Description and Calibration Procedure

**Figure 1:**
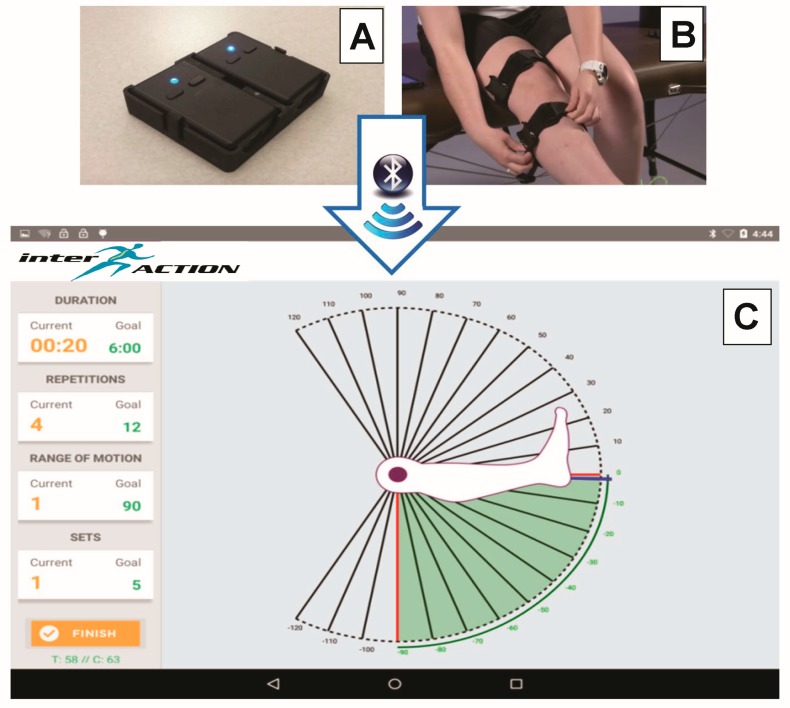
(**A**) Yost Lab’s two 3-Space Bluetooth sensors is a 3D printed case designed to align the sensors during alignment, (**B**) Padded elastic straps secured on the thigh and shank, Cary, (**C**) Screenshot of the mobile application screen that provides the participant with visual feedback.

The portable IMU-based motion tracking system consists of two IMUs (3-Space Bluetooth Sensor, Yost Labs, Portsmouth, OH, USA), each composed of a tri-axial accelerometer, gyroscope, and magnetometer sensors (Figure 1A). Although the IMUs were factory calibrated and provided with default values for the accelerometer and magnetometer, they were re-calibrated in house to account for local magnetic disturbances and to determine the gyroscope parameters. Each IMU sensor was individually calibrated prior to data collection using the Yost Lab’s 3-Space Sensor Software Suite’s 24-step gradient descent calibration procedure to account for the scale, bias, and cross-axis effects [20]. The 24-step gradient descent calibration procedure required that the sensor is oriented in 24 unique orientations; therefore, a custom (non-ferrous) calibration jig was constructed to ensure each orientation was accurately implemented. 

Upon successful calibration, the IMUs provide an orientation estimation using the manufacturer provided Kalman filter. The Kalman filter utilizes normalized sensor data and reference vectors to optimally combine the data into a final orientation reading in quaternion format. The individual IMUs have a manufacturer stated orientation accuracy of ±1 degree for dynamic conditions across all orientations, an orientation resolution of <0.08 degree, and an orientation repeatability of 0.085 degree. Further technical details about the 3-Space sensors and their sensor fusion algorithms are discussed here [21,22] and can be found on the manufacturer’s website.

### 2.2. Video-Based Motion Tracking System Description and Calibration Procedure 

A six-camera video-based motion tracking system (OptiTrack, NaturalPoint, Corvallis, OR, USA) running the Motive: Tracker software was utilized as the “gold standard” [23,24]. Prior to data collection, a triangulation procedure was utilized to associate the position of known calibration markers (CWM-125, NaturalPoint) from each of the six synchronized cameras. The ground plane and origin of the video-based motion tracking system was established using a 200 mm calibration square (CS-200, NaturalPoint). The average overall wand error calculated during the calibration procedure was <0.1 mm. After successful calibration, the Motive:Tracker software was utilized to stream the *xyz* position of 4 mm reflective markers that were adhered directly to the corners of the IMU cases. The marker position data was utilized to define a local coordinate system for each sensor and the orientation to be calculated and reported in quaternion format. 

### 2.3. Measurement of Knee Joint Angles

Knee movement was recorded simultaneously by the IMUs and OptiTrack. To detect the joint angle of the knee, the two IMUs were fastened to the participant’s leg using elastic straps (Mueller Sports Medicine, Prairie du Sac, WI, USA) and custom 3D printed sensor cases. The IMUs were fastened equidistant from the middle of the patella, the first was secured to the thigh and the second was secured to the shank (Figure 1B). Since the IMUs were mounted to the irregular contours of the human body, it was also necessary to account for the offset between the sensors location and the anatomical coordinate system. There are several published methods of determining the anatomic coordinate system [25], however for this project a static manual anatomical alignment approach was utilized, which was adapted from previously published approaches [26,27,28]. This approach was selected to ensure all users regardless of functional status (e.g., early post-operative rehabilitation following a total knee replacement) were capable of successfully completing the anatomical alignment procedure independently. The static manual alignment procedure was adapted from methods described previously [27,28] and was implemented as follows. The IMUs were first placed in a sensor alignment tool (Figure 1A), which was manually aligned to the subject’s anatomy. This orientation was recorded as the reference orientation. The subject then removed the IMUs from the alignment tool and placed them in the sensor cases secured to their thigh and shank. The difference between the orientation on the thigh and shank relative to the reference orientation was calculated and utilized to transform the data into an anatomical coordinate system. 

The anatomically aligned movement of the thigh and shank were recorded in quaternions, and the knee joint motion was calculated as quaternion difference of the shank relative to the thigh. The data was reported using Euler angles, which were calculated using Euler angle decomposition assuming flexion-extension, varus-valgus, and internal-external order of rotation to avoid gimbal lock. 

### 2.4. interACTION Application

When interACTION is used clinically, the patient’s treating physical therapist provides a personalized exercise program (from a library of more than 30 routinely prescribed exercises) and offers interactive guidance to the patient through a clinician portal. The patient can access the prescribed exercise regimen remotely in the mobile application. Prior to performing their exercises, the mobile application guides the patient through a series of recovery metrics (RoM, straight leg raise, and pain score) to quantitatively assess their recovery progress. For each exercise that has been prescribed, a written description and video demonstration is provided to educate the patient on the proper execution of the exercise. After the patient is confident in the purpose and proper execution of the intended exercise, they begin the exercise and use the interface to complete the number of repetitions and sets prescribed by the physical therapist. The motion data collected by the mobile application is analyzed and displayed in a customizable, joint-specific manner to provide real-time feedback to the patient, and quantitatively monitor the patient’s adherence. The real-time qualitative and quantitative visual feedback is displayed using a two-dimensional animation and a numerical counter depicting the knee joint angle (Figure 1C). A color-coded mechanism informs the patients if they are outside (red), approaching (yellow), or reached (green) the individualized RoM target. Using predefined thresholds, which are consistent with the color-coded feedback, the system automatically counts the number of repetitions completed and categorizes them according to the quality of the repetition (attempted repetitions versus successful repetitions). The recovery and adherence data collected in the mobile application is wireless synchronized to the clinician portal for remote management using a cloud-based database (Microsoft Azure). 

### 2.5. Data Collection

A convenience sample of ten healthy control participants (males: *n* = 7, females: *n* = 3, age: 23.8 ± 3.6 years old, weight: 158.7 ± 28.2 pounds, height: 68.4 ± 3.1 inches) were recruited for an Institutional Review Board approved protocol (IRB PRO14110431). Participants had to be able to walk on a treadmill for at least ten minutes and lack a history of significant knee pain, injury, surgery, or dysfunction. 

Under supervision of the research coordinator, the participants secured the sensors to their legs using elastic straps and performed an automated anatomical alignment procedure. Participants were directed to perform ten repetitions of three knee rehabilitation exercises at a self-selected, comfortable pace: heel slides (0° to 60° flexion), short arc quadriceps (SAQ) contractions (0° to 30°) and sit-to-stand (0° to 90° flexion). The heel slide exercise was first completed without feedback from the mobile application, and then all exercises were performed using visual feedback from the mobile application. To ensure that the participant understood the target RoM for the heel slide without visual feedback, the research coordinator demonstrated the angle using a goniometer. 

After concluding the exercises, the participants completed a custom usability survey about their experience with interACTION. The custom survey included fourteen questions covering the following topics: user interface (four questions), usability (three questions), wearability (five questions), and aesthetics (two questions). Participants were asked to rate each question using a five-point Likert scale that ranged from 0 (lowest or worst) to 4 (highest or best). Participants also completed the After-Scenario Questionnaire (ASQ), a validated measure of user satisfaction for ease of use, amount of time needed, and clarity of instruction on a seven-point Likert scale that ranged from 1 (strongly disagree) to 7 (strongly agree) [29]. 

### 2.6. Data Analysis

The kinematic data recorded by the IMUs and OptiTrack was processed using MATLAB (The MathWorks, Inc., Natick, MA). The datasets between the IMU and Optitrack measurements were aligned in time by making the first peak flexion angle the first data point in the analysis. RoM was calculated as the difference between the maximum and minimum flexion-extension angle for each repetition. During the analysis it was determined that a procedural error was made during the data collection for one participant; therefore, that participant’s kinematic data was excluded from the final statistical analyses. Statistical analyses were conducted using SAS version 9.4 (SAS Institute Inc., Cary, NC, USA).

#### 2.6.1. Variability and Accuracy

The mean ± standard deviation (SD) RoM was calculated and reported to assess the intra-subject variability. The data was displayed graphically using Bland-Altman Plots, to evaluate the differences between the IMU and OptiTrack to determine the relationship between measurement error and the true value. To assess the accuracy of the IMUs, root mean squared error (RMSE) was calculated relative to OptiTrack. Intraclass correlation coefficients (ICC_2,1_) were also calculated in order to assess the consistency (or reproducibility) between the IMUs and OptiTrack. The following scale was used for interpretation of the ICC values: 0–0.49 = poor agreement, 0.5–0.74 moderate agreement, 0.75–0.89 good agreement, 0.9–1.0 excellent agreement [30]. 

#### 2.6.2. Visual Feedback

To assess the effect of visual feedback on exercise performance, the heel slides with visual feedback and the heel slides without feedback were compared with the prescribed target RoM value of 60 degrees using a one-sample t-test (* *p* < 0.05). The data was also displayed graphically using a scatterplot of the difference between the target RoM value of 60 degrees and the actual RoM for the heel slides with visual feedback, and the heel slides without feedback.

#### 2.6.3. Survey Data

The median and mode of the custom usability survey and ASQ data were calculated, and to ensure transparency the response frequency was also reported. 

## 3. Results

### 3.1. Variability and Accuracy

**Figure 2:**
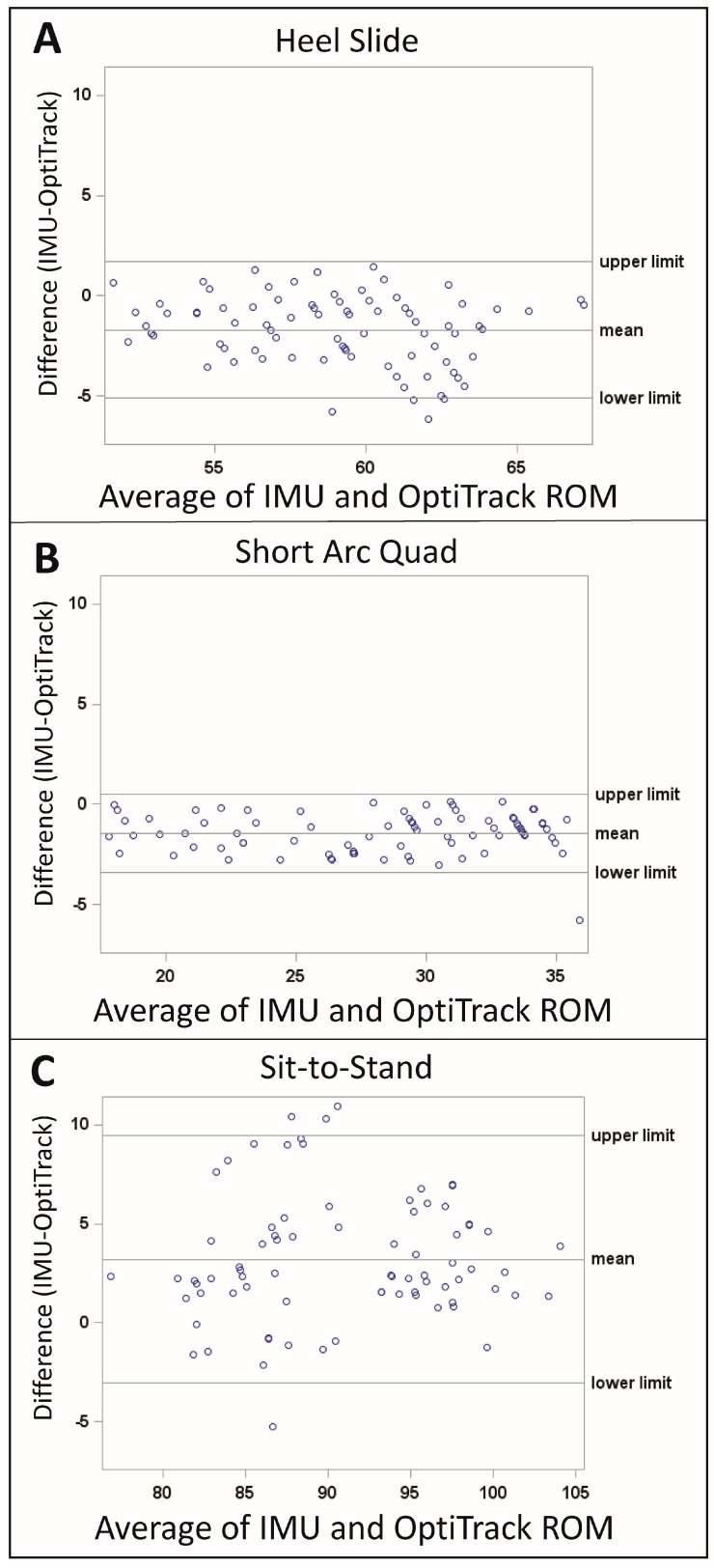
Bland-Altman Plots displaying the differences between the IMU and OptiTrack for the (**A**) heel slide, (**B**) SAQ, and (**C**) sit-to-stand exercises.

The differences between the IMU and OptiTrack data is displayed in Figure 2. For the heel slide the mean difference was −1.7 degrees, and the limits of agreement were −5.1 degrees and 1.7 degrees (Figure 2A). For the SAQ, the mean difference was −1.4 degrees, and the limits of agreement were −3.4 degrees and 0.5 degrees (Figure 2B). The sit-to-stand exercise had the largest RoM, and correspondingly the largest mean difference (3.2 degrees) and limits of agreement (−3.0 degrees and 9.5 degrees) (Figure 2C).

The mean ± SD RoM for the IMUs and the OptiTrack was inclusive of the prescribed RoM for all exercises performed: heel slides (58.2 ± 3.5 degrees), SAQ (27.5 ± 5.3 degrees), and sit-to-stand (92.5 ± 6.7 degrees) (Table 1). The intra-subject variability was 2.5 degrees or less for all exercises, and the sit-to-stand exercise had the highest variability of the exercises performed.

The RMSE values of the IMUs, when compared with those of OptiTrack, were less than three degrees; the sit-to-stand had the largest RMSE (2.9 degrees), followed by the heel slides (2.4 degrees), and the SAQ (2.0 degrees). The ICCs for all exercises indicated a moderate (heel slides = 0.58) to good agreement (SAQ = 0.86 and sit-to-stand = 0.80) between the IMUs and OptiTrack.

### 3.2. Visual Feedback

**Figure 3:**
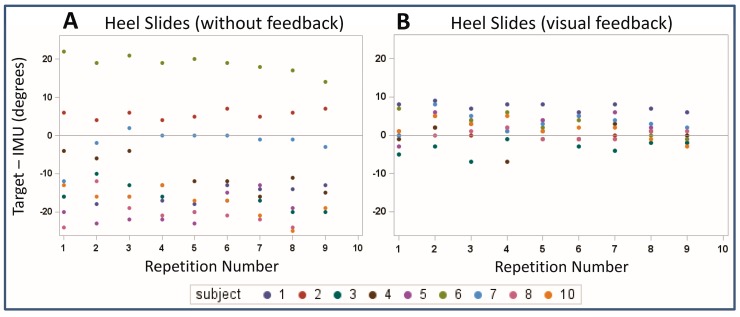
Scatterplot of the difference between the target ROM value of 60 degrees and the actual ROM for (**A**) the heel slides without visual feedback and (**B**) the heel slides with visual feedback.

The difference between the target RoM of 60 degrees and the actual RoM recorded by the IMUs ranged between approximately −20 degrees and 20 degrees with no visual feedback (Figure 3A), but with visual feedback this range decreased to −5 degrees to 10 degrees (Figure 3B). 

When no visual feedback was provided to the participant performing the heel slides, the mean RoM was approximately 10 degrees larger than the prescribed RoM target of 60 degrees (70.0°, 95% CI: 59.0° to 80.9°, *p* = 0.07). When the participant was given visual feedback using the interACTION system, the 95% CI for the mean RoM was dramatically reduced and centered on the RoM target (mean RoM 60.1°, 95% CI 57.1° to 63.1°, *p* = 0.93).

### 3.3. Survey Data

**Figure 4:**
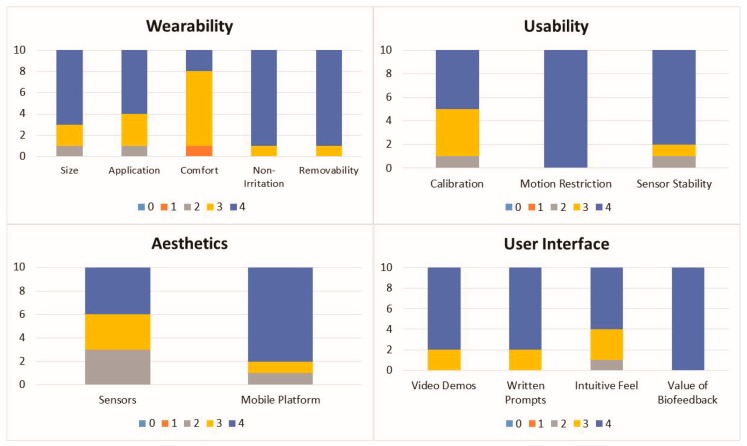
Frequency bar graphs of the custom usability survey results.

The response frequency for the usability survey is shown in Figure 4. The median and mode values of all of the usability survey responses were ≥3 and 11 out of the 14 questions had the maximum median value of 4 and the maximum mode value of 4.

**Figure 5:**
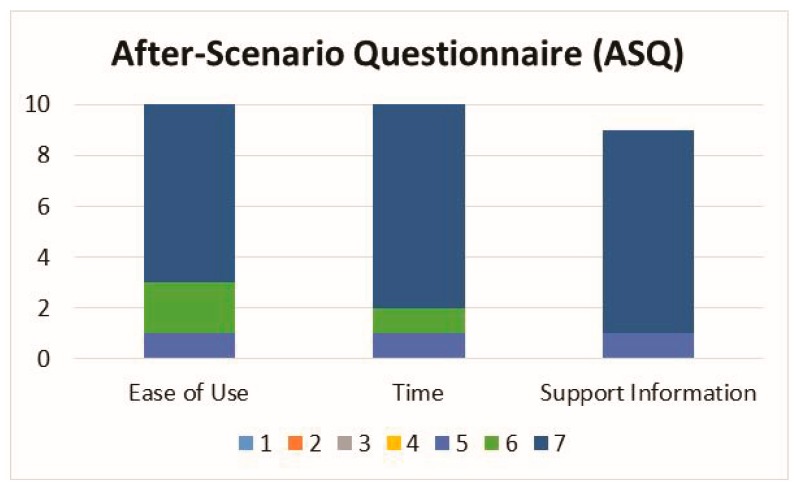
Frequency bar graph of the ASQ survey results.

The three questions that had median or mode values lower than 4 were wearability: comfort (median = 3, mode = 3), aesthetics: sensors (median = 3, mode = 4), and usability: calibration (median = 3.5, mode = 4). The response frequency for the ASQ is shown in Figure 5. The median and mode values for the ASQ were equal to 7 for all survey questions. 

## 4. Discussion

The IMU-based knee angle measurement system provided an accurate and repeatable measure of human knee joint motion during physical therapy exercises. Validation with OptiTrack showed that the IMU measurements had an RMSE of less than three degrees of the “gold standard”, with moderate to good correlation between the two for all measured exercises. The Bland-Altman mean difference ranges from −1.7 to 3.2 degrees and the limits of agreement were the largest for the SAQ exercise (−3.0 degrees and 9.5 degrees). These results are consistent with previous reports that have used optical tracking systems, electromagnetic tracking systems, or potentiometers as benchmarks [27,31,32,33,34,35,36,37].

More specifically, Leardini et al. performed a study that evaluated the use of IMUs for knee flexion, extension, lunge, and squat exercises, and reported a mean difference of 3.9 to 5 degrees [17]. Picerno et al. evaluated flexion-extension and walking tasks, and reported an error ranging from 0.2 degrees to 2.9 degrees [25]. Tognetti et al. also looked at flexion and walking, and found a mean error of 2.0 degrees [38]. Tulipani evaluated a total of six functional tasks and reported an error of less than 5 degrees [35]. Similarly, Al-Amri evaluated walking, squatting, and jumping, and also reported an error of less than 5 degrees, and Jaysrichai et al. reported an error of less than 6 degrees [39,40]. The most directly comparable study to our present study was reported by Liu et al., wherein they evaluated the sit-to-stand exercise using a wearable sensor system [41]. Liu et al. concluded that inertial sensors are able to monitor the sit-to-stand exercise with a high degree of accuracy and have potential for rehabilitation training. 

In the present study, the differences between the IMU and OptiTrack measurements were also displayed graphically in the Bland-Altman plots. Observation of these plots for each exercise did not demonstrate a relationship between measurement error and the true value. However, between exercises it was observed that as the RoM of the exercise increased, the measurement error between the IMUs and the OptiTrack increased. This relationship was also apparent in the RMSE parameters. One possible explanation for the increased error with increasing RoM is that the static anatomical alignment method implemented in this study did not fully align the IMUs to the anatomy. Future work should explore if implementation of a dynamic alignment method [25] would reduce the measurement error and explore the reliability of the alignment methods in the target patient population. 

The most unique aspect of this study was its design to quantify the effect of visual feedback on exercise performance. Visual feedback significantly improved the participant’s ability to achieve the prescribed motion target, indicating that IMUs can monitor both the completion and quality of the exercises. This is consistent with previous reports that have shown the benefits of coupling feedback with the use of IMUs; however, the majority of the previous literature focused on measurement of gait and balance, rather than specific rehabilitation exercises [42,43,44,45,46,47,48]. In balance and gait stability Bechly et al. compared the effects of visual and vibrotactile feedback on exercise performance [42]. The study focused on adherence and typical lack of exercise feedback as a deterrent for patients. The authors’ hypothesis is that feedback can improve performance, which leads to greater confidence in exercises and more consistency in performing exercises. Similarly, Ginis et al. validated a rehabilitation tool with feedback, in that it uses IMUs available to users in their smartphones in combination with an app for measuring movement [44]. The device is intended to test balance in Parkinson’s patients. Ginis et al. validated the device (CuPiD) accuracy and usability in several clinical exams typically prescribed to Parkinson’s patients. Similar to the present study, the RCT focused on validation for use in the home and provided realtime feedback for each exercise. The accuracy of performance with feedback was one of the primary outcome measures. Vadnerkar et al. focused on rehabilitation exercises targeting gait improvement [47]. The IMUs used are contained in a module attached to the patient’s foot, and data is collected via Bluetooth connection and stored on a smartphone. Similarly, the device incorporates biofeedback into exercise execution. The study saw favorable outcome with effective measurement of accuracy in the prescribed exercise (heel-to-toe) [47].

Based on the custom usability survey results, interACTION was well received by the study participants. Survey results also helped identify areas for improvement (e.g., comfort, sensor aesthetics, and the calibration procedure) prior to testing the system in a patient population. A limitation of the custom survey is that it is difficult to compare to the literature. Therefore, a validated measure of satisfaction (ASQ) was also collected, which demonstrated that the participants were highly satisfied with the interACTION system. 

Although this study was able to sufficiently answer the proposed research questions, there were limitations that should be considered when interpreting the findings. The sample size was small and one participant’s data had to be excluded due to technical difficulties experienced during set-up. However, this sample size is consistent with similar studies [33,35]. The population tested, consisting entirely of young, healthy volunteers, was appropriate for this study’s aims but may prevent the findings from being directly translated to older populations with acute or chronic conditions. Although there are several conditions (i.e., anterior cruciate ligament reconstruction), which are commonly performed in an age group consistent with the population tested in this manuscript, it is recommended that the usability of the system is reevaluated for each patient population of interest. When interpreting the impact of the visual feedback, it is also important to note that the heel slides without feedback were always completed before the heel slides with feedback. Although this is a limitation of the study, it is consistent with the patients’ experience when performing their exercises at home without the oversight of a physical therapist. Although the physical therapist may provide verbal or visual feedback in the clinical setting, when the patient performs their exercises at home, they typically do not have access to any feedback mechanism. If we had reversed or randomized the order of testing, it would have artificially reduced the difference between the target and actual RoM for the simulated home exercise. 

In order to translate interACTION into the clinical setting, this study demonstrated the platform’s ability to measure RoM during rehabilitation exercises for physical therapy and that visual feedback significantly improved the participant’s ability to perform the exercises correctly. The participants of this study were also highly satisfied with the wearability, usability, and overall look and feel of the technology as well as the ease of use, amount of time needed, and clarity of instruction. Future studies will utilize interACTION to examine its impact on post-surgical physical therapy outcomes such as cost-savings, effects on adherence, and quality of life. We also plan to expand the use of IMU motion tracking to other joints such as the hip, shoulder, and spine. With the ultimate goal of reducing unnecessary healthcare costs and improving patient outcomes, this system will allow us to offer a convenient remote therapy option for patients who may experience barriers with traditional physical therapy.

## Figures and Tables

**Table 1:** Data from IMUs and OptiTrack system demonstrating the accuracy and variability.

	Heel Slides	Short Arc Quad	Sit-to-Stand
RoM IMUs (Mean ± SD)	58.2° ± 3.5°	27.5° ± 5.3°	92.5° ± 6.7°
RoM OptiTrack (Mean ± SD)	59.9° ± 3.9°	28.9° ± 5.3°	89.3° ± 6.5°
Intra-Subject Variability IMUs	2.4°	2.0°	2.5°
RMSE (IMUs vs. OptiTrack)	2.4°	2.0°	2.9°
ICC (IMUs vs. OptiTrack)	0.58	0.86	0.80
